# British Association for the Advancement of Medical Science

**Published:** 1856-03

**Authors:** 


*British Association for the Advancement of Medical Science.*—This association has had several very interesting meetings at Glasgow during the past week, attended by Baron Liebig, Dr. Daubeny, Dr. Playfair, Sir David Brewster, Dr. Carpenter, Dr. Stenhouse, Professor Owen, etc. etc. Amongst the few subjects in relation to medicine, we may mention that Dr. Daubeny, of Oxford, exhibited a set of small grain weights, for weighing medicines, made of the new metal "aluminium," for which it seems peculiarly adapted, by its very superior brilliance as a metal, and its great lightness: so that $\frac{1}{8}$th of a grain of strychnine, for instance, may be weighed with a weight as large as the present $\frac{1}{2}$ grain weight of the surgery drawer. The aluminium weight also does not contract rust or verdigris. A new compound of chlorine, analogous to the allotropic condition of oxygen in ozone, was exhibited by Dr. Andrews, of Belfast, and may hereafter be found to play an important part in the theory of disinfection and deodorization by chlorine. Baron Liebig read a paper on a new compound of fulminic acid, the salts of which are of adamantine brilliancy. Chevalier Clauson exhibited artificial gutta percha, likely to be very useful in forming splints and other appliances, for surgeons. Various interesting papers were also communicated, on the physiological changes in marine vivaria, etc.—*Lancet*, Sept. 22, 1855.—*Med. News.*—*Counselor.*

